# Supplementary material for: Biosynthetic constraints on amino acid synthesis at the base of the food chain may determine their use in higher-order consumer genomes
Source: PLoS Genet. 2023 Feb 13;19(2):e1010635. doi: 10.1371/journal.pgen.1010635 (PMC9956874; doi:10.1371/journal.pgen.1010635)
Supplement: S1 Table — (PDF) [file pgen.1010635.s001.pdf]

Holidic Food - 1L				
Product	Amount	Unit	Company	Code
Agar	2.00E+01	g	Sigma-Aldrich	A7002-1KG
Sucrose	1.71E+01	g	Sigma-Aldrich	S1888-5KG
Acetic Acid	3.00E+00	mL	Thermo-Fisher	AJA1-2.5L GL
KH2PO4	3.00E+00	g	Sigma-Aldrich	P9791-500G
NaHCO3	1.00E+00	g	Sigma-Aldrich	S8875-500G
CaCl2	2.50E-01	g	Sigma-Aldrich	C7902-500G
MgSO4	2.50E-01	g	Sigma-Aldrich	M7506-500G
CuSO4	2.50E-03	g	Sigma-Aldrich	C7631-250G
FeSO4	2.50E-02	g	Sigma-Aldrich	F7002-250G
MnCl2	1.00E-03	g	Sigma-Aldrich	M3634-100G
ZnSO4	2.50E-02	g	Sigma-Aldrich	Z0251-100G
Choline Chloride	5.00E-02	g	Sigma-Aldrich	C1879-1KG
Myo-inositol	5.00E-03	g	Sigma-Aldrich	I7508-100G
Inosine	6.50E-02	g	Sigma-Aldrich	I4125-10G
Uridine	6.00E-02	g	Sigma-Aldrich	U3750-25G
Thiamine	1.41E-03	g	Sigma-Aldrich	T4625-5G
Riboflavin	6.93E-04	g	Sigma-Aldrich	R4500-5G
Nicotinic Acid	8.38E-03	g	Sigma-Aldrich	N4126-100G
Ca Pantothenate	1.08E-02	g	Sigma-Aldrich	21210-5G-F
Pyridoxine	1.74E-03	g	Sigma-Aldrich	P9755-25G
Biotine	1.41E-04	g	Sigma-Aldrich	B4501-1G
Folic Acid	5.00E-04	g	Sigma-Aldrich	F7876-1G
Propionic Acid	6.00E+00	ml	Merck	8.00605.0500
Nipagin	7.50E-01	g	Sigma-Aldrich	W271004-5KG-K
Cholesterol	3.00E-01	g	Glentham	GE0100
Absolute Ethanol*	3.00E+01	mL	Thermo-Fisher	AJA214-2.5LPL
Amino Acids	1.07E+01	g	See AA ratio table	
*Solvent for cholesterol and nipagin				

SY food - 1L				
Product	Amount	Unit	Company	Code
Agar	1.00E+01	g	Gelita	A-181017
Graded Sugar	5.00E+01	g	Bundaberg	M180919
Brewer's Yeast	1.00E+02	g	MP Biomedicals	903312
Nipagin	1.50E+00	g	Sigma-Aldrich	W271004-5KG-K
Propionic Acid	3.00E+00	ml	Merck	8.00605.0500
Absolute Ethanol*	1.50E+01	mL	Thermo-Fisher	AJA214-2.5LPL
*Solvent for nipagin				

Amino Acids (10.7g/L) - 1L				
FLYAA				
AA	Amount	Units	Company	Code
ESSENTIALS			ESSENTIALS	
F	5.04E-01	g	Sigma-Aldrich	P2126-100G
H	3.27E-01	g	Sigma-Aldrich	H8000-100G
I	5.61E-01	g	Sigma-Aldrich	I2752-100G
K	6.82E-01	g	Sigma-Aldrich	L5626-100G
L	1.02E+00	g	Sigma-Aldrich	L8912-100G
M	3.01E-01	g	Sigma-Aldrich	M9625-100G
R	8.14E-01	g	Sigma-Aldrich	A5131-100G
T	5.53E-01	g	Sigma-Aldrich	T8625-100G
V	5.99E-01	g	Sigma-Aldrich	V0500-500G
W	1.60E-01	g	Sigma-Aldrich	T0254-100G
NON-ESSENTIALS			NON-ESSENTIALS	
A	5.50E-01	g	Sigma-Aldrich	A7627-100G
C	1.71E-01	g	Sigma-Aldrich	C7477-100G
D	5.85E-01	g	Sigma-Aldrich	A6683-100G
E	7.59E-01	g	Sigma-Aldrich	G5889-500G
G	3.83E-01	g	Sigma-Aldrich	G7126-100G
N	5.14E-01	g	Sigma-Aldrich	A0884-100G
P	4.88E-01	g	Sigma-Aldrich	P0380-100G
Q	5.60E-01	g	Sigma-Aldrich	G3126-100G
S	6.88E-01	g	Sigma-Aldrich	S4500-100G
Y	4.64E-01	g	Sigma-Aldrich	T8566-100G

MALEAA			FEMALEAA		
AA	Amount	Units	AA	Amount	Units
ESSENTIALS			ESSENTIALS		
F	5.04E-01	g	F	4.69E-01	g
H	2.80E-01	g	H	2.90E-01	g
I	1.12E+00	g	I	1.08E+00	g
K	8.86E-01	g	K	9.65E-01	g
L	1.88E+00	g	L	1.78E+00	g
M	2.96E-01	g	M	2.75E-01	g
R	8.30E-01	g	R	9.04E-01	g
T	4.99E-01	g	T	5.13E-01	g
V	5.78E-01	g	V	5.83E-01	g
W	1.82E-01	g	W	1.68E-01	g
NON-ESSENTIALS			NON-ESSENTIALS		
A	5.49E-01	g	A	5.43E-01	g
C	2.01E-01	g	C	2.43E-01	g
D	6.58E-01	g	D	6.88E-01	g
E	7.74E-01	g	E	8.12E-01	g
G	3.96E-01	g	G	3.99E-01	g
N	4.86E-01	g	N	4.78E-01	g
P	4.66E-01	g	P	4.42E-01	g
Q	5.02E-01	g	Q	5.29E-01	g
S	6.14E-01	g	S	6.07E-01	g
Y	8.40E-01	g	Y	8.45E-01	g
