# Supplementary material for: Biosynthetic constraints on amino acid synthesis at the base of the food chain may determine their use in higher-order consumer genomes
Source: PLoS Genet. 2023 Feb 13;19(2):e1010635. doi: 10.1371/journal.pgen.1010635 (PMC9956874; doi:10.1371/journal.pgen.1010635)
Supplement: S2 Table — (PDF) [file pgen.1010635.s002.pdf]

FLYAA

		per litre (g)	mM	ratio
Essential amino acids				
phenylalai	F	1.01	6.11	0.04
histidine	H	0.65	4.22	0.03
isoleucine	I	1.12	8.55	0.05
lysine	K	1.37	9.34	0.06
leucine	L	2.03	15.50	0.09
methionin	M	0.60	4.04	0.02
arginine	R	1.63	9.37	0.06
threonine	T	1.11	9.30	0.06
valine	V	1.20	10.25	0.06
tryptopha	W	0.32	1.57	0.01
Non-essential amino acids				
alanine	A	1.10	12.36	0.07
cysteine	C	0.34	2.82	0.02
aspartate	D	1.17	8.81	0.05
glutamate	E	1.52	10.32	0.06
glycine	G	0.77	10.22	0.06
asparagin	N	1.03	7.80	0.05
proline	P	0.98	8.50	0.05
glutamine	Q	1.12	7.67	0.05
serine	S	1.38	13.11	0.08
tyrosine	Y	0.93	5.13	0.03
		21.39		

MALEAA

		per litre (g)	mM	ratio
Essential amino acids				
phenylalaı	F	1.07	6.47	0.04
histidine	H	0.60	3.82	0.02
isoleucine	I	1.21	9.17	0.06
lysine	K	1.51	10.29	0.06
leucine	L	2.02	15.31	0.09
methionin	M	0.63	4.22	0.03
arginine	R	1.46	8.36	0.05
threonine	T	1.06	8.88	0.05
valine	V	1.23	10.44	0.06
tryptopha	W	0.39	1.89	0.01
Non-essential amino acids				
alanine	A	1.17	13.07	0.08
cysteine	C	0.38	3.14	0.02
aspartate	D	1.08	8.05	0.05
glutamate	E	1.43	9.69	0.06
glycine	G	0.85	11.20	0.07
asparagin	N	1.03	7.79	0.05
proline	P	0.99	8.58	0.05
glutamine	Q	1.07	7.29	0.04
serine	S	1.31	12.38	0.08
tyrosine	Y	0.90	4.97	0.03
		21.39		

FEMALEAA

		per litre (g)	mM	ratio
Essential amino acids				
phenylalai	F	1.00	6.02	0.04
histidine	H	0.62	3.96	0.02
isoleucine	I	1.17	8.86	0.05
lysine	K	1.64	11.20	0.07
leucine	L	1.90	14.47	0.09
methionin	M	0.58	3.91	0.02
arginine	R	1.59	9.09	0.06
threonine	T	1.09	9.12	0.06
valine	V	1.24	10.53	0.06
tryptopha	W	0.36	1.75	0.01
Non-essential amino acids				
alanine	A	1.15	12.92	0.08
cysteine	C	0.31	2.59	0.02
aspartate	D	1.12	8.41	0.05
glutamate	E	1.50	10.16	0.06
glycine	G	0.85	11.27	0.07
asparagin	N	1.02	7.67	0.05
proline	P	0.94	8.13	0.05
glutamine	Q	1.12	7.67	0.05
serine	S	1.29	12.23	0.07
tyrosine	Y	0.91	5.00	0.03
		21.39		

Amino Acid	3-letter Code	1-letter Code	Molecular Weight (g/mol)
Alanine	Ala	A	89.1
Cysteine	Cys	C	121.2
Aspartate	Asp	D	133.1
Glutamate	Glu	E	147.1
Phenylalan	Phe	F	165.2
Glycine	Gly	G	75.1
Histidine	His	H	155.2
Isoleucine	Ile	I	131.2
Lysine	Lys	K	146.2
Leucine	Leu	L	131.2
Methionine	Met	M	149.2
Asparagine	Asn	N	132.1
Proline	Pro	P	115.1
Glutamine	Gln	Q	146.2
Arginine	Arg	R	174.2
Serine	Ser	S	105.1
Threonine	Thr	T	119.1
Valine	Val	V	117.1
Tryptophan	Trp	W	204.2
Tyrosine	Tyr	Y	181.2

**FLYAA**	**stock**	
	**200**	ml
ESSENTIALS		
F	3.33	g
H	2.16	g
I*		
K	4.51	g
L*		
M	1.99	g
R	5.38	g
T	3.65	g
V	3.96	g
W	1.06	g
NON-ESSENTIALS		
A	3.64	g
C**		
D	3.87	g
E***		
G	2.53	g
N	3.40	g
P	3.23	g
Q	3.70	g
S	4.55	g
Y*		

**MALEAA**	**stock**	
	**200**	ml
ESSENTIALS		
F	3.33	g
H	1.85	g
I*		
K	5.86	g
L*		
M	1.96	g
R	5.49	g
T	3.30	g
V	3.82	g
W	1.20	g
NON-ESSENTIALS		
A	3.63	g
C**		
D	4.35	g
E***		
G	2.62	g
N	3.21	g
P	3.08	g
Q	3.32	g
S	4.06	g
Y*		

**FEMAL EAA**	**stock**	
	**200**	ml

ESSENTIALS

F	3.10	g
H	1.92	g
I*		
K	6.38	g
L*		
M	1.82	g
R	5.97	g
T	3.39	g
V	3.85	g
W	1.11	g

NON-ESSENTIALS

A	3.59	g
C**		
D	4.55	g
E***		
G	2.64	g
N	3.16	g
P	2.92	g
Q	3.50	g
S	4.01	g
Y*		

common solutions **stock**

	**200**	ml	
E	20	g	makes 100mg/ml stock (If using the free acid, this will only be soluble by adding NaOH)
C	10	g	makes 50mg/ml stock

individually to bottle at start of each cook (before autoclaving). See example recipes for final additions

** for most ratios, cys precipitates out over time. Best to omit from stock and add to medium as a separate (50mg/ml) soln.

*** E is added separately. Done for flexibility so if an amino acid or a group of amino acids are omitted, E can be adjusted to compensate for loss of N.

- these solutions are close to saturation so patience is required!

- adjust final pH of aa solutions using NaOH or HCl to about pH 4.5 (note that if using glutamate (free acid), it will only go into solution at the indicated conc with NaOH)

- filter sterilise into sterile tubes or bottles

- store at room temp or 4C away from light

- add to media after autoclaving

FLYAA

	1000 ml
total mass of amino acids	**21.4g**

agar		20	g
ile		1.12	g
leu		2.03	g
tyr		0.93	g
sucrose		17.12	g
cholesterol		15	ml
buffer		100	ml
CaCl2	1000x	1	ml
MgSO4	1000x	1	ml
CuSO4	1000x	1	ml
FeSO4	1000x	1	ml
MnCl2	1000x	1	ml
ZnSO4	1000x	1	ml
total		805.97	
nucl/lipid soln		8	ml
stock solution	EAA	60.51	ml
stock solution	NEAA (w/o cys)	60.51	ml
stock solution	glutamate (100mg/ml)	15.19	ml
stock solution	cys (50mg/ml)	6.83	ml
vit		21	ml
folic acid		1	ml
prop acid		6	ml
nipagin		15	ml

MALEAA

	1000 ml
total mass of amino acids	**21.4g**

agar		20	g
ile		2.24	g
leu		3.76	g
tyr		1.68	g
sucrose		17.12	g
cholesterol		15	ml
buffer		100	ml
CaCl2	1000x	1	ml
MgSO4	1000x	1	ml
CuSO4	1000x	1	ml
FeSO4	1000x	1	ml
MnCl2	1000x	1	ml
ZnSO4	1000x	1	ml
total		802.78	
nucl/lipid soln		8	ml
stock solution	EAA	60.51	ml
stock solution	NEAA (w/o cys)	60.51	ml
stock solution	glutamate (100mg/ml)	15.48	ml
stock solution	cys (50mg/ml)	9.72	ml
vit		21	ml
folic acid		1	ml
prop acid		6	ml
nipagin		15	ml

FEMALEAA

	1000 ml
total mass of amino acids	**21.4g**

agar		20.00	g
ile		2.16	g
leu		3.55	g
tyr		1.69	g
sucrose		17.12	g
cholesterol		15.00	ml
buffer		100.00	ml
CaCl2	1000x	1.00	ml
MgSO4	1000x	1.00	ml
CuSO4	1000x	1.00	ml
FeSO4	1000x	1.00	ml
MnCl2	1000x	1.00	ml
ZnSO4	1000x	1.00	ml
total		803.72	
nucl/lipid soln		8.00	ml
stock solution	EAA	60.51	ml
stock solution	NEAA (w/o cys)	60.51	ml
stock solution	glutamate (100mg/ml)	16.24	ml
stock solution	cys (50mg/ml)	8.02	ml
vit		21.00	ml
folic acid		1.00	ml
prop acid		6.00	ml
nipagin		15.00	ml
