# Supplementary figures and images for: Biosynthetic constraints on amino acid synthesis at the base of the food chain may determine their use in higher-order consumer genomes
Source: PLoS Genet. 2023 Feb 13;19(2):e1010635. doi: 10.1371/journal.pgen.1010635 (PMC9956874; doi:10.1371/journal.pgen.1010635)

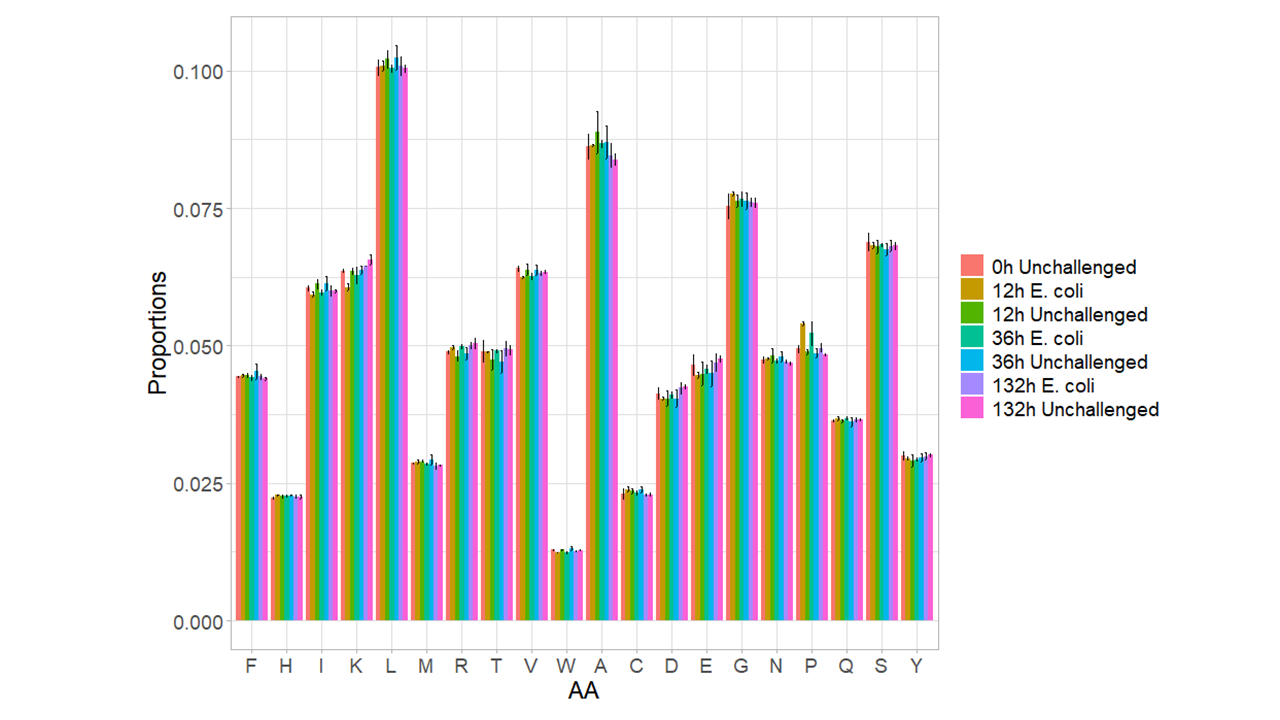

Supplement: S1 Fig — (TIF) [file pgen.1010635.s003.TIF]

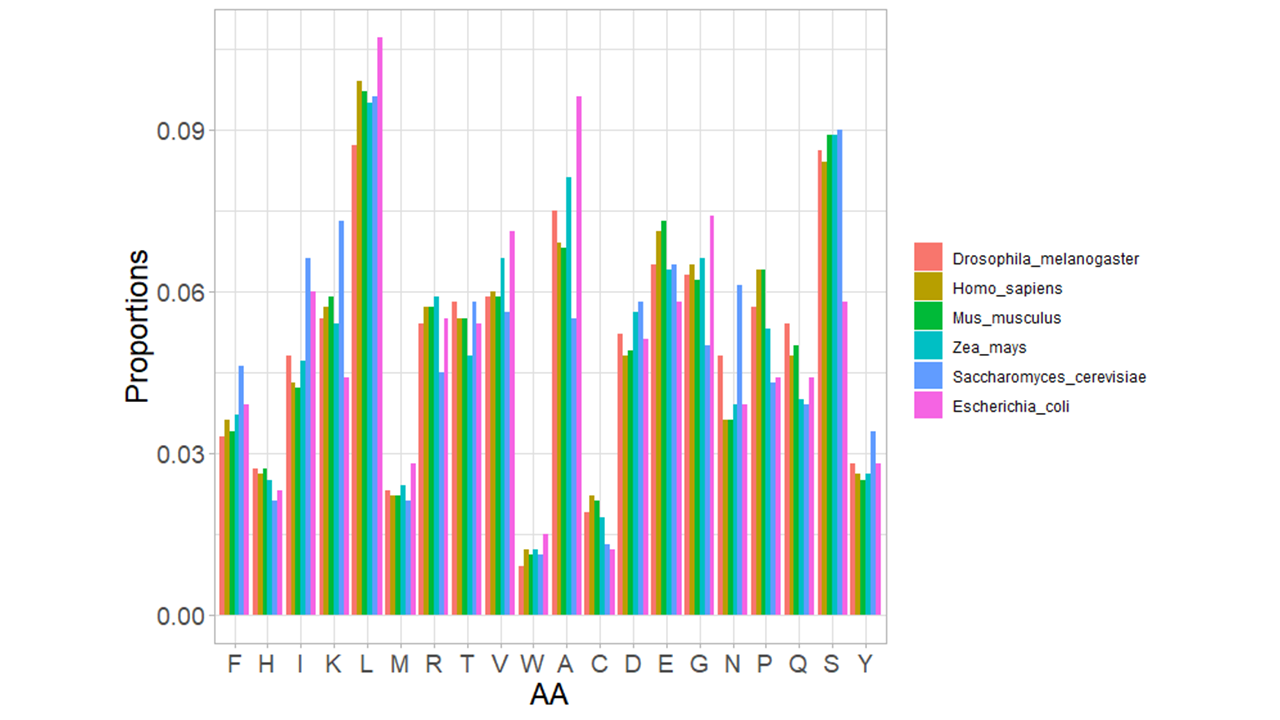

Supplement: S2 Fig — (TIF) [file pgen.1010635.s004.TIF]
