# Supplementary material for: Biosynthetic constraints on amino acid synthesis at the base of the food chain may determine their use in higher-order consumer genomes
Source: PLoS Genet. 2023 Feb 13;19(2):e1010635. doi: 10.1371/journal.pgen.1010635 (PMC9956874; doi:10.1371/journal.pgen.1010635)
Supplement: Attachment — Submitted filename: Gomez Ortega_cover & responses_221228.docx [file pgen.1010635.s006.docx]

*Dear Dr Copenhaver*

*Many thanks for overseeing the re-revision of our manuscript, and for the helpful comments with useful advice. We have now addressed these comments (see below) and adjusted our manuscript accordingly. We hope you now find this is appropriate for publication in PLoS Genetics.*

*All the very best*

*Matt*

Dear Dr Piper,

Thank you very much for submitting your Research Article entitled 'BIOSYNTHETIC CONSTRAINTS ON AMINO ACID SYNTHESIS AT THE BASE OF THE FOOD CHAIN MAY DETERMINE THEIR USE IN HIGHER-ORDER CONSUMER GENOMES' to PLOS Genetics.

The manuscript was fully evaluated at the editorial level and by two of the original independent peer reviewers. One reviewer is now satisfied (but notes a couple of minor typos).  The other reviewer still has fundamental concerns about the first half of the manuscript.  If possible, addressing their concerns experimentally would be optimal, but if that is not feasible, I believe it would be sufficient to explicitly state the limitations/caveats of the conclusions drawn from that data in the text of the manuscript (as described by the reviewer) - that would allow readers to weight those conclusions appropriately.

*The referee raises an important point - we cannot rule out that some type of transcriptome weighted exome matching could yield a positive outcome(s) for some other aspect(s) of health and fitness that we did not measure. However, further experiments to test other metrics of health may not help to address this, because fitness is a composite of numerous trait values and it is not feasible to measure them all. The possibility will thus always remain that transcriptome-weighted diets could benefit one or more unmeasured traits. For instance, if we tested infection responses and saw no benefits, it would still not demonstrate that transcriptome weighted exome matching may or may not be beneficial for predator avoidance, or in other different contexts. We therefore chose to measure traits that are closely related to fitness, and commonly used as a proxies for fitness, and which our previous experiments have shown are sensitive to dietary amino acid profile.*

*We have added some text to the end of the discussion to address this important point,*

Reviewer's Responses to Questions

**Comments to the Authors:
Please note here if the review is uploaded as an attachment.**

Reviewer #2: The authors have addressed all of my comments. Two minor typos I saw:

L42: Double “that”

L50: “protein limitation”: i.e., not plural

*Both of these errors have been corrected.*

Reviewer #3: I have re-read the manuscript and unfortunately I do not feel that the revisions adequately address the last round of comments.

The second half of the paper (biosynthetic constraints of AA production) is publishable alone in PLoS genetics, independent of the first half of the paper. However the first half fundamentally delivers a null result, but since the assays of males and females are different, we don't know if lack of response to transcriptome weighting is a true negative - we might just be measuring different things. For this reason an assay that can be conducted equivalently before and after transcriptome weighting would be more appropriate. Assays of immunity before and after weighting would indeed be very interesting, and I recognise the authors' efforts to include some data on this. However without functional immune assays to show whether flies fed weighted/unweighted diets are more resistant to infection, we cannot make any conclusions. Showing that immune response-weighted diets are predicted to be less different than sex-weighted diets does not mean that the assays of males and females reproduction are definitely measuring the same thing.

My recommendation is still that the second half of the paper is a solid publishable unit, but the first half does not convince me that transcriptome-weighted diets should not be pursued.

i. *Why include the negative data (first half of the manuscript)?*

*Probably the most frequently asked question about exome matching is whether weighting the information by expression levels can yield an improvement in its ability to predict the amino acid requirements of the consumer. This is an important question with implications for both fundamental science and commercial applications. Using an example with extreme differences, we find that there is no evidence to support that transcriptome weighting the exome is beneficial. Because this is interesting and important, we think that the negative outcomes in the first part of the manuscript are worth reporting. However, because the negative results that we report do not rule out its potential advantages in other settings, we have added some text to the end of the discussion to leave the possibility open.*

ii. *Should we have measured “fitness” differently?*

*Proxies of fitness can be measured in many ways and we needed to choose a trait to measure. In both sexes we standardised the comparison by choosing what is commonly and justifiably considered the trait most closely related to the physiological determinant of fitness, fecundity. We obviously couldn’t measure the same thing in both sexes, but we did take precautions to ensure that each assay was sensitive to dietary amino acid levels. This is particularly well known for females for whom our past work has shown a strong predictive relationship between the level of the most limiting dietary essential amino acid and egg laying* [1]*. Thus, even if it is not possible to compare changes in female fitness with changes in male fitness in response to diet, which was not our intention, it is still possible to compare the effect of diet on a fitness metric in each sex.*

iii. *Should we have measured something else?*

*Measuring the response of female egg laying to dietary amino acids is currently the trait that is most well understood and justified by the literature* [1–3]*. Measuring another trait, such as response to infection, could yield a different outcome to what we observed for fecundity. But if the particular type of infection assay we employed did not, it would still not demonstrate that transcriptome weighted exome matching may or may not be beneficial in other different contexts. Therefore, while we agree with the reviewer that the infection weighted exome data do not reveal that the male and female fitness assays were equivalent (this was not the point), they do show that male and female-weighted exome data diverge more from exome matching than infection-weighted data. We use this to argue that, of all assays possible, that which we employed had the strongest evidence to support that we might detect an effect.*

**References**

1 Piper MDW, Soultoukis GA, Blanc E, Mesaros A, Herbert SL, Juricic P, He X, Atanassov I, Salmonowicz H, Yang M, Simpson SJ, Ribeiro C & Partridge L (2017) Matching Dietary Amino Acid Balance to the In Silico-Translated Exome Optimizes Growth and Reproduction without Cost to Lifespan. *Cell Metab* 25, 610–621.

2 Ma C, Mirth CK, Hall MD & Piper MDW (2020) Amino acid quality modifies the quantitative availability of protein for reproduction in Drosophila melanogaster. *J Insect Physiol*, 104050.

3 Piper MDW, Blanc E, Leitão-Gonçalves R, Yang M, He X, Linford NJ, Hoddinott MP, Hopfen C, Soultoukis GA, Niemeyer C, Kerr F, Pletcher SD, Ribeiro C & Partridge L (2014) A holidic medium for Drosophila melanogaster. *Nature Methods* 11, 100–5.
