# Supplementary material for: Biosynthetic constraints on amino acid synthesis at the base of the food chain may determine their use in higher-order consumer genomes
Source: PLoS Genet. 2023 Feb 13;19(2):e1010635. doi: 10.1371/journal.pgen.1010635 (PMC9956874; doi:10.1371/journal.pgen.1010635)
Supplement: Attachment — Submitted filename: Gomez Ortega et al_cover & responses to reviewer comments.pdf [file pgen.1010635.s005.pdf]

Dear Dr Copenhaver,

Please consider this revised submission of our manuscript PGENETICS-D-22-00575.

In response to the very helpful comments from the reviewers we have made some significant changes to the manuscript that have greatly improved it. In doing so, we have invited Prof David Raubenheimer to contribute as an author for his expertise in nutritional ecology.

Together with the reviewer comments, we decided to extensively modify the latter part of the article. This has resulted in us removing Figures 5 and 6, introducing two new supplementary figures, and re-writing the discussion. These changes, and the reasons for them, are described in detail below in the responses to reviewer comments.

We hope you find this revised version of the manuscript to be as interesting as what we do; we describe a striking fundamental new finding that supply-side constraints on amino acid production in autotrophs appears to shape the biomass composition of all organisms, from bacteria to apex predators.

Yours sincerely

Matthew Piper

Head of the Nutritional Physiology Lab
School of Biological Sciences,
Monash University

Comments to the Authors:

Please note here if the review is uploaded as an attachment.

Reviewer #1:

This manuscript represents an original study which tests whether the exome or the transcriptome is a better predictor of the amino acid requirements of adult Drosophila. The authors predicted that the transcriptome should be a better match to the amino acid requirements of specific sexes of flies than the average Drosophila 'exome'. The manuscript calculates the amino acid requirements based on published transcriptomes from male and female flies. The authors test whether fecundity – the ultimate fitness measure - is influenced by the concentration of amino acids and the profile derived from the exome or the transcriptome. They found that the concentration of the amino acids available had a strong influence on the ability of males to fertilize females and of females to lay eggs. The transcriptome based diet, however, did not improve performance. They also identified the role of specific amino acids in the performance of males (tryptophan) and females (lysine). I found this experiment a fascinating extension of their previous work and a valuable contribution to the literature.

The authors also compared the AAs of 53 organisms including autotrophs (plants) and heterotrophs (animals, unicellular organisms). They concluded that animals had less diverse AA profiles than plants. They also concluded that 'extremophiles' were more constrained than 'nonextremeophiles'. While I thought this was interesting, I also thought that Figures 5 and 6 were the weakest part of the manuscript.

I suggest that the analysis of the AAs of the exomes of the species shown in Figure 5 is conducted in a way that is a better fit with the rest of the manuscript. The data in this graph is quite biased towards heterotrophs, and it does not look like a linear model fits these data very well. I think that the authors should also provide more of a justification for the species chosen in this analysis. They need to better balance the representation of phyla and perhaps compare heterotrophs and autotrophs that are multicellular (or according to some other specific logic), rather than including unicellular organisms and comparing only heterotrophs and autotrophs. An analysis which did more to compare Drosophila to other insects and then to other animals would actually fit better in the context of the manuscript than this broad analysis of heterotrophs and autotrophs.

I also had difficulty believing the analysis of the data presented in Figure 6. Again, there is a strong data bias in the 'non-extremophile' group relative to the 'extremophile' group. This section also lacks a strong justification, and though the authors cite Krick et al. 2014 as the basis for the study of the metabolic cost of production of amino acids, I found it lacking enough detail for me to follow the arguments made in this paper.

In response to these very helpful comments, we have decided to remove the latter analysis and Figures 5 and 6. Instead, we draw more heavily on the excellent work of Krick et al, 2014 (ref 41) to expand the discussion. In doing so, we have introduced two new supplementary figures:

Suppl fig 1. To show the transcriptome weighted exome match data for infected and uninfected flies. This is introduced to show how broadly AA proportions are conserved across other situations where the flies are challenged (i.e. how resistant the AA proportions are to changing from the exome match predictions despite large scale expression changes)

Suppl fig 2. A cross species comparison of AA proportions, including the following organisms: bacteria, yeast, mouse, fly, human and corn. This figure is not intended to be an exhaustive comparison of AA profiles and their costs (since this is covered well in Krick et al, 2014 for bacteria)[1], but instead conveys the finding that despite enormous evolutionary distance between bacteria and humans, the relative proportion of AA represented in their exomes is similar. This extends the work of Lightfield et al [2], who found a similar conservation across distantly related bacterial phyla.

Since these two figures are novel findings, but largely support the discussion, we have chosen to include them as supplementary figures

Minor comments:
How many samples were used to calculate the transcriptome profiles used in these experiments?

This information can be found in the methods section:

- Five transcriptomes for each diet
- Three transcriptomes for each tissue
- Three transcriptomes for each timepoint after each E. coli infection. (new data for supplementary figure 1)

Were these transcriptomes of single flies or composites from pooled samples?

Unfortunately, this information is not specified in the data sources, but given the date of collection, it is very likely these were from pooled samples. We refer to them in the methods as “male samples” and “female samples”.

Were several transcriptomes used, and at what time point in the animal’s life history were they collected?

This information can be found in the methods section. The data from ModEncode is the product of duplicates and those from FlyAtlas, and the newly included infection study, are from triplicates.

These details are missing from the manuscript but they are important to the interpretation of the results.

lines 390-393 - I don't follow the logic that heterotrophy 'constrains' evolution by limiting genome AA usage. Surely a counter example is aphids, which suck sap that is deficient in essential amino acids, and acquire needed amino acids from symbionts.

We have now modified the language in the discussion to be more careful. We try to convey that the constraints of nutrient availability result in evolved strategies to overcome those limitations. These include, but are not limited to, associations with microbes, selective nutrient stores, as well as selective digestion and absorption of nutrients. Despite these strategies, the proportion of AA in distantly related organisms’ proteomes remains similar. This indicates that even those organisms that employ the strategy of having microbial associations to overcome AA limitations, like aphids and termites, they are still only able to attain AA in the proportions that their symbionts can supply. Thus, the limitations on autotrophs still constrains the AA proportions in heterotrophs.

Reviewer #2:

In this paper the authors report the effects of transcriptome-weighted exome matching on reproductive functioning in Drosophila. Relative to regular exome matching transcriptome weighting does not significantly improve reproductive function in either sex. Based on this result the authors argue that the efficiency of the use of dietary amino-acids is constrained at some pre-transcription level of biology. A cross-taxa synthesis reveals that the exome-matched composition of diets differs more among heterotrophs than autotrophs, and that the energetic costs of synthesising amino-acids explains their relative requirements.

This is an interesting paper that has been well prepared for publication. The paper reads well and guides the reader through the logic of the different steps in the analysis. It makes clever use of data. I have some comments that the authors may wish to take into consideration. Generally, I am supportive of publication.

L120-122. Are the authors at all concerned about the imprecision/specificities of the expression data they are using? Presumably the gene expression data used are an estimate from an experiment, which come with standard errors etc and will have been measured in different animals. I know the authors can only use what is available and I am not suggesting they should have done anything else, but I think this is a point that should perhaps be addressed in the discussion.

This is a good point. We have now introduced error bars to indicate the variation in transcriptome weighted exome match profiles to illustrate the variability in the transcriptome data. These represent only a small fraction of the mean and do not affect the predicted limiting AA.

We also note that this issue is partially addressed by predictions from the tissue specific AA profiles (panel F in figures 2 and 3). In each case, the sex-matched profiles are predicted to be better matched than exome matching alone, even though the identity of the predicted limiting AA may change with the tissue used. Thus, if variability in the whole-body transcriptomic data has produced an inaccurate prediction in the identity of the limiting AA for the flies, the sex-matched AA profile should still be sufficiently improved over exome matching to produce better performance.

L189. “another diet in which tryptophan was omitted” – was this the rich FLYAA diet with tryptophan removed? If so, I would specify that, if not, what was it?

Yes, this was the rich FLYAA diet with tryptophan removed. We describe this now in the results section.

L211. RE salivary glands, could this be an alternative explanation for your results? If both MALEAA and FEMALEAA are deficient for the salivary glands is food intake impacted on these diets, meaning that in fact intake of amino-acids is impacted relative to FLYAA?

We have now added a phrase to the results to explicitly acknowledge this caveat. There is limited knowledge of the function of the adult salivary glands, but they are thought to aid in ion balance and not in digestion as is the case for mammalian saliva. However, even if they were critical for protein digestion, they should not be required for our flies feeding on synthetic food since we add each AA as a purified ingredient, which therefore does not require digestion.

L352-354. This is a neat way of seeing how the exome-matched diets compare in context. I wondered whether adding the composition of yeast also helps add this context? For reference, for example, are all three exome matched diets in Fig 1 more similar to one another than to some commonly used yeast (e.g., in the SY diet)?

We agree that this is useful context. We have now added the yeast (*Saccharomyces cerevisiae*) exome match profile, alongside profiles from other species (including flies) in Fig S2. These data show that the three fly profiles used in our study are more similar to each other than the yeast is to fly.

L376. I found the wording of this sentence a bit strange. Seems more natural to say "… the transcriptome affects/dictates the consumer's AA requirements."

We have changed this in line with the reviewer's suggested wording.

Figure 5. This looks like regression through the origin here. You need to be very careful that these models pass a validation step. Often regression through the origin makes sense biologically, but violates model assumptions. In the case of analysis its often better to sacrifice the former in favour of the latter.

This plot has now been removed from the manuscript. Instead, we have expanded the discussion to explain the regression data generated by Krick et al, 2014 [1].

L405-413. Two points here. 1) Revealing my ignorance about how the cost of calculation is made, here, but isn't the differential cost to extremeophiles (e.g., due to temperature) factored in, or is a common method used? 2) It would be nice if it were possible to include a directional prediction? Do expect all extremophiles to differ in the same direction?

This is a great point.

Because this part of the manuscript is now more based in the excellent analytical and computational work that others have done (eg Lightfield et al, Krick et al, Chen et al, Akashi et al, Mazel et al, Baudouin-Cornu et al. [1–6]) we have moved this part to the discussion and this particular prediction has been modified. Instead of focusing on extremophiles, we note a more generalised prediction that terrestrial animals will be methionine limited, and the evidence that flies have evolved to buffer against this limitation.

Figure 6. This regression/scatter plot seems upside down to me.

In restructuring this part of the manuscript, have now removed this plot (please see above).

L503-512. I really struggled with this, even after having read it several times. I suspect may other readers would as well. Can the authors bring more clarity to what is being conveyed.

In restructuring this part of the manuscript, this section has been rewritten to broaden the point beyond flies. We hope the point is now made more clearly.

Reviewer #3:

This manuscript by Ortega et al concerns the match between dietary amino acid supply and user need, using a Drosophila model. This is the latest in a string of interesting and important papers from the authors in this area.

The paper comes in two halves. The first half builds on published results showing that in silico translation of the exome predicts a ratio of dietary amino acids that better matches consumer need. In the current manuscript, the authors ask if weighting the exome match algorithm with transcriptome data can improve the prediction of optimal diet. This extension of the published protocol is not successful in the current study, with results showing that diets matched to sexual dimorphism in the transcriptome do not enhance the fitness of either sex. The second half of the paper goes on to show that in silico translation of exomes of organisms across higher trophic levels yields patterns of amino acid use that are predicted by costs of biosynthesis in primary producers. Throughout, data appear to have been collected at the highest standard, analysis is appropriate, writing is clear, and dataviz is outstanding.

The second half of the paper is extremely interesting, novel, and has potentially major implications for our understanding of how nutritional physiology and ecology evolve. I believe this second half alone would attract significant interest if published in PLoS Genetics. Indeed the paper's title refers only to this half. I have only one question about this part of the paper, for discussion: if AA usage regresses to cost of production, there should be close relationship between AA ratios across organisms. Why then in previous work was yeastAA shown to be sub-optimal relative to flyAA? Is the difference between these organisms basically residual variation away from the mean(s)?

Organisms have evolved physiological and behavioural mechanisms to buffer against some variability in dietary AA supply. However, this buffering capacity is limited. Beyond this limit, fitness becomes compromised. We propose that the yeast profile (supplementary figure 1) is sufficiently different from the flies' requirements so as to create a physiological cost. By contrast, the transcriptome weighted exome match diets (Figure 1) are similar enough to the fly's requirements that they can continue to sustain egg production at an unchanged rate.

My enthusiasm for the first half of the paper is somewhat more limited: it is not clear what these new results add to the field, and the paper's title does not mention them. Basically, the implicit conclusion is that exome matching doesn't work, but previous results show unambiguously that it can. Transcriptome-weighted exome matching might work in other contexts, e.g. stress resistance. This means that the second part of the paper, which does contain new findings, is not well set up by the first, and indeed the jump from matching diets to transcriptomes is a bit sudden, without progressive logic. It is clearly important to be able to publish negative results, and it's clear that a lot of work went into these experiments, but in this case without some forms of positive controls,

some alternative explanations for the (unfortunate) lack of signal are possible. My questions are:

Could males and females just be so different that fitness assays are not comparable? Might sex-matched diets improve other traits that could be measured in equivalently in each sex but weren't measured here? e.g. xenobiotic or starvation resistance?

We have tried to tackle this by including another transcriptome-weighted exome match profile from a physiological challenge that is thought to be protein demanding and elicit large transcriptional responses: infection. These profiles (Supplementary figure 1) show that weighting the exome values by the infected transcriptome produces even less difference in the AA proportions than when weighting by the sex-specific transcriptomes. Thus, we propose that the sex differences have given us the greatest chance to detect a change in dietary AA requirements.

Why use female insemination and egg production assays?

We know that female egg production is extremely sensitive to dietary AA levels. In past work, we have shown an almost perfect correspondence between exome match predictions of female egg production and actual egg production (Piper et al, *Cell metabolism*, 2017) [7], a fact that is further supported by the response of female egg production to lysine dilution (Fig 3D). Thus, even though the assay for males in our manuscript is unproven (although see our negative control data in Fig 2A and Fig 2E that support a role for protein at the level of single essential AA) and appears to have less power to detect differences between AA profiles, our data show that female egg production is highly sensitive to AA profiles.

Thus, we conclude that it is not possible to include a positive control because our current control (exome matched) is the positive control for measurable aspects of fly fitness. It is possible that other traits are affected, but it is difficult to know what else to test as there is no good hypothesis to test traits beyond the reproductive metrics we measured.

Are the FlyAtlas data sufficiently granular to reveal the reproduction-limiting cells in each sex - Do we know which reproductive tissue is protein-limited? FlyAtlas characterized many tissues, but there is still a lot of uncharacterized complexity within a tissue, and it can take only a very few cells to limit overall function.
Might the sexes even differ in which tissues are limiting? e.g. could male fitness not be a function of courtship and therefore function of particular neurons, which might not limit females in the same way?
The male fitness assay measures number of inseminated females, which is influenced by behavior. Might number of offspring per inseminated female have been a more direct measure of sperm quality, indicative of nutritional quality?
The FlyAtlas data were collected in different flies, on a different diet. Both the male and female transcriptome were likely different from what they would be on

FLYAA, which is the real baseline we would need for transcriptome weighting to improve upon. Might confounding variation have clouded impact of transcriptome weighting?

Please see our previous comment as it speaks to this same point.

In summary, I enjoyed reading the paper, I believe it will be of interest to the readership of PLoS Genetics, and will make an important impact on multiple fields. However I think the important parts aggregate in the second half.

References:

1. Krick T, Verstraete N, Alonso LG, Shub DA, Ferreiro DU, Shub M, et al. Amino acid metabolism conflicts with protein diversity. Mol Biol Evol. 2014;31: 2905–2912. doi:10.1093/molbev/msu228
2. Lightfield J, Fram NR, Ely B. Across bacterial phyla, distantly-related genomes with similar genomic GC content have similar patterns of amino acid usage. PLoS One. 2011;6: e17677. doi:10.1371/journal.pone.0017677
3. Chen Y, Nielsen J. Yeast has evolved to minimize protein resource cost for synthesizing amino acids. Proc Natl Acad Sci U S A. 2022;119: e2114622119. doi:10.1073/pnas.2114622119
4. Akashi H, Gojobori T. Metabolic efficiency and amino acid composition in the proteomes of Escherichia coli and Bacillus subtilis. Proc Natl Acad Sci U S A. 2002;99: 3695–3700. doi:10.1073/pnas.062526999
5. Mazel D, Marlière P. Adaptive eradication of methionine and cysteine from cyanobacterial light-harvesting proteins. Nature. 1989;341: 245–248. doi:10.1038/341245a0
6. Baudouin-Cornu P, Surdin-Kerjan Y, Marlière P, Thomas D. Molecular evolution of protein atomic composition. Science (80-). 2001;293: 297–300. doi:10.1126/science.1061052
7. Piper MDW, Soultoukis GA, Blanc E, Mesaros A, Herbert SL, Juricic P, et al. Matching Dietary Amino Acid Balance to the In Silico-Translated Exome Optimizes Growth and Reproduction without Cost to Lifespan. Cell Metab. 2017;25: 610–621. doi:10.1016/j.cmet.2017.02.005
